# Impact of Base Rubber and Cure Systems in Additive Manufacturing of Fully Compounded Thermoset Elastomers

**DOI:** 10.3390/polym18040540

**Published:** 2026-02-23

**Authors:** AA Mubasshir, Stiven Kodra, Chandramouli Sangeetham, David O. Kazmer, Joey L. Mead

**Affiliations:** 1 Department of Plastics Engineering, University of Massachusetts, Lowell 01854, MA, USA; david_kazmer@uml.edu (D.O.K.); joey_mead@uml.edu (J.L.M.); 2 Department of Mechanical Engineering, University of Massachusetts, Lowell 01854, MA, USA; stiven_kodra@student.uml.edu (S.K.); chandramouli_sangeetham@student.uml.edu (C.S.)

**Keywords:** nitrile butadiene rubber (NBR), ethylene propylene diene monomer (EPDM), thermoset elastomer, additive manufacturing, 3D printing, curing agents, sulfur cure, peroxide cure, interlayer adhesion, shrinkage

## Abstract

While the effects of formulation variables of a rubber compound are well established for conventional rubber manufacturing techniques, their role in extrusion-based additive manufacturing remains underexplored. This study explores the impact of different base rubbers (NBR and EPDM) and curing agents (sulfur and peroxide) on processability and final part characteristics in material extrusion additive manufacturing applications. Under identical printing conditions, sulfur-cured NBR exhibits greater post-print shrinkage (12%) than sulfur-cured EPDM (7%). However, sulfur-cured NBR achieves a higher degree of adhesion between printed layers than sulfur-cured EPDM, as suggested by the % retention of the bulk materials’ ultimate stress by the printed parts (84–100% and 51–62%, respectively). Additionally, a peroxide-cured NBR formulation was compared against the same sulfur-cured NBR formulation. Printed parts from the peroxide-cured NBR formulation showed higher shrinkage (16%) and lower % retention of the bulk materials’ ultimate stress (26–33%) than the sulfur-cured NBR formulation. Additionally, the tensile behavior of all three rubber compounds was found to be strongly dependent on printing orientation, showing the anisotropic behavior typical of extrusion-based additive manufacturing. Sulfur-cured NBR showed the least anisotropy for stress at break (0.82) and strain at break (0.90), whereas peroxide-cured NBR showed the highest anisotropy in stress (0.74) and strain (0.82). The anisotropy ratios for sulfur-cured NBR and EPDM compounds were very similar for stress (0.82 vs. 0.82) and comparable for strain (0.90 vs. 0.87). Notably, the peroxide cure system provided almost twice as much available printing time as the sulfur cure system. This report on the effects of base rubber and curing agents on 3D printability and part properties provides a background to guide future efforts to design rubber compounds for 3D printing applications.

## 1. Introduction

The term “elastomer” refers to a class of polymeric materials capable of undergoing large, reversible deformations due to their network of long, polymeric chains interconnected by either physical or chemical crosslinks [1]. Based on the nature of these crosslinks, elastomers can be broadly categorized into two classes: thermoplastic elastomers (TPEs) and thermoset elastomers (TSEs). While TPEs typically exhibit thermally reversible physical crosslinks. TSEs (commonly known as rubbers) possess permanent chemical crosslinks [2,3,4]. Irrespective of the nature of crosslinks, elastomeric materials are increasingly attracting interest in additive manufacturing (AM) due to their unique combination of elasticity, resilience, and durability, which enables applications requiring energy absorption, sealing, vibration damping, and flexible load bearing.

Additive manufacturing of thermoplastic elastomers (TPEs) is dominated by thermoplastic polyurethanes (TPUs), commonly processed via fused deposition modeling (FDM), where printability is governed by melt viscosity, elastic recovery, and interlayer diffusion during deposition [5,6,7,8,9]. Beyond TPU, styrene block copolymers [10], polyamide elastomers [11], and copolyester elastomers [12] have also been reported. Other 3D printing methods used for TPEs are direct ink writing (DIW) [13,14,15] and powder bed fusion [16,17]. Across these studies, anisotropic mechanical behavior, particularly reduced properties along the build direction, is frequently highlighted as a defining limitation. In contrast, chemically crosslinked elastomers present distinct challenges and opportunities, as their final properties arise from post-deposition crosslink formation rather than melt solidification. Recent reviews categorize thermoset elastomer 3D printing strategies into three categories, namely photopolymerization-based systems, reactive two-component extrusion, and heat-activated extrusion of curable rubber compounds [18]. Among these, stereolithography (SLA)-based 3D printing of photopolymers has been the dominant one, with research concentrated in silicone rubber, rubber-like resins and photocurable polyurethane resin [18,19,20]. Among synthetic rubber grades used extensively in the rubber industry, styrene–butadiene rubber (SBR) was printed via vat photopolymerization by Scott et al. [21], isoprene rubber via digital light processing (DLP) by Strohmeier et al. [22], ethylene propylene diene monomer (EPDM) via vat photopolymerization by Wen et al. [23], and both polybutadiene rubber and butyl rubber via direct ink writing (DIW) by Bragaglia et al. [24] and Shen et al. [25], respectively. While these studies demonstrate the feasibility of printing industrially relevant synthetic rubbers in photocurable formulations, commercial applications largely depend on heat-activated crosslinking systems. Additive manufacturing of heat-curable synthetic rubber formulations remains comparatively underexplored, and the present study aims to address this gap with particular emphasis on base rubber and cure chemistry.

The use of thermally crosslinkable synthetic rubber compounds in the additive manufacturing process is very recent. Unlike thermoplastic materials, rubbers are seldom used in their virgin form. Rubber is generally used as a compound of multiple chemical additives (base rubber, curing agents, accelerators, accelerator activators, reinforcing fillers, antidegradants, processing aids, and other miscellaneous ingredients) to improve the processability and properties of the base rubber [26,27,28]. Such practices make rubber compounds a challenging material class to process in additive manufacturing. On the one hand, an uncured rubber compound is too viscous for AM techniques such as polymer jetting; on the other hand, filaments made from uncured rubber lack the required stiffness to be used in fused filament fabrication methods [29]. Recent developments of screw- and ram-driven extrusion mechanisms for printing compounded rubbers have allowed more detailed studies of this material class’s behavior in 3D printing applications [30,31]. For example, Sundermann et al. [32] showed the effects of a liquid NBR on the cure kinetics and mechanical anisotropy of 3D-printed parts. Sundermann et al. [33] also reported the effects of a scorch retarder to extend the processing window of an NBR formulation designed for additive manufacturing. Previous work by this group explored the use of a commercial plasticizer as a viscosity modifier to tackle the issue of post-print shrinkage in rubber 3D printing [34]. Although such an approach proved effective in reducing the shrinkage, adverse effects on the mechanical properties of printed parts were noticed. However, no literature was found on the effects of base rubber and curing agents on overall printability, mechanical properties, quality of the adhesion among neighboring roads of printed parts, and available processing window in 3D printing applications.

Changing one component of a rubber compound usually has multiple effects on the compound’s processability and final product properties. Hence, proper designing of a rubber compound for any processing technique requires characterization of the effects of each component of the rubber compound on the processability and properties of the final product. Although the effects of base rubber and curing agents on compression- and injection-molded parts are well studied, due to the nascent nature of the rubber AM technology, such a study has not been conducted yet. The base rubber and curing system play dominant roles in determining processing and final material properties [26,27,28]. In this study, two industrially important synthetic rubbers, nitrile butadiene rubber (NBR) and ethylene propylene diene monomer rubber (EPDM), were investigated under identical material extrusion AM conditions. In addition to base rubber chemistry, the two most widely employed heat-activated crosslinking approaches in the rubber industry, sulfur- and peroxide-based curing systems, were also evaluated. From an additive manufacturing perspective, differences in base rubber, cure kinetics, scorch safety, and crosslink structure may significantly affect printability, inter-road adhesion, and mechanical performance of printed parts. To shed light on such effects, in this work, we investigated the effect of different base elastomers and cure systems on the processing and tensile behavior in additive manufacturing. Their behavior was compared against compression-molded parts.

Two key objectives guided the investigation. The first objective aimed to establish a comparative understanding of NBR and EPDM formulations in 3D printing applications. To achieve this, we formulated sulfur-cured NBR and EPDM compounds with identical ingredients and quantities. The resulting materials were then evaluated for post-print shrinkage, available processing window, and the tensile behavior of 3D-printed parts. This comparison allowed for a direct assessment of the inherent printability and part properties of two significant synthetic rubbers, NBR and EPDM. The second objective focused on the impact of curing agents on the printability, available processing window, and tensile properties of an NBR compound. Here, we compared sulfur-cured NBR to a peroxide-cured NBR formulation. Both compounds contained the same ingredients and quantities, with the sole exception being the curing agent. The subsequent evaluation of post-print shrinkage, printing window, and tensile behavior in the 3D-printed parts from each formulation shed light on the influence of the chosen curing method on the printability and final properties of NBR.

## 2. Materials and Methods

### 2.1. Materials

The base elastomers, nitrile butadiene rubber (NBR) and ethylene propylene diene monomer (EPDM), were used in two different forms in this study, a low and a high molecular weight version. The high-molecular-weight NBR (Nipol DN2835, ZEON Chemicals, Louisville, KY, USA) had a Mooney viscosity (ML 1 + 4 at 100 °C) of 30–40, and the low-molecular-weight NBR, referred to as the low-MW NBR (Nipol 1312, ZEON Chemicals, Louisville, KY, USA), had a Mooney viscosity of 10–20. The high-molecular-weight EPDM (Royalene 511, LION Elastomers, Geismar, LA, USA) had a Mooney viscosity (ML 1 + 4 at 100 °C) of 45, and the low-molecular-weight EPDM, referred to as the low MW EPDM (Trilene 65, LION Elastomers, Geismar, LA, USA), had a Brookfield viscosity of 177000 cps at 100 °C. Commercial-grade N330 carbon black (CB) with a particle size of 28–36 µm was used as reinforcing filler (N330 carbon black, Tokai Carbon CB Ltd., Fort Worth, TX, USA). N-cyclohexyl benzothiazole-2-sulfenamide, or CBS (DURAX (CBS) powder, Vanderbilt Chemicals LLC, Norwalk, CT, USA), was used as the accelerator. Commercial-grade zinc oxide (ZOCO102^®^, Zochem Inc., Brampton, ON, Canada) and stearic acid (A292-500^®^, Fisher Chemical, Ottawa, ON, Canada) were used as accelerator activators. A commercial dicumylperoxide (DiCup^®^ R/Luperox^®^ DCP dicumyl peroxide, Arkema Inc., Bristol, PA, USA) and commercial sulfur (Rubber Maker, Harwick Standard, Akron, OH, USA) were used as the vulcanizing agents.

### 2.2. Preparation of Rubber Compounds

The formulations of all three rubber compounds are reported in Table 1. NBR-PO was a peroxide-cured NBR formulation, NBR-Sul was a sulfur-cured NBR formulation, and EPDM-Sul was a sulfur-cured EPDM with 60 PHR carbon black, along with conventional additives commonly employed in NBR formulations, as detailed in Table 1. Compounding or mixing of formulation ingredients was performed in 50 g batches with a Brabender Plasticorder Intelli-Torque Plus (Model 01-55-000, Duisburg, Germany) internal mixer with counter-rotating screws. The ingredients were added to the mixture in the order listed in Table 1. The mixing procedure consisted of kneading the rubber for 2 min, followed by the addition of carbon black and mixing for an additional 5 min to ensure proper dispersion of CB particles. Next, activators, accelerators, and curing agents were added in the order shown in Table 1. After the addition of the curing agent, the batch was mixed until the mixing torque became stable, indicating good mixing had been achieved. The total mixing time for all compounds was 20 min.

In this article, material formulations are henceforth denoted using abbreviations of the base rubber and the corresponding curing agent. Peroxide and sulfur curing systems are abbreviated as PO and Sul, respectively. Accordingly, the peroxide-cured NBR compound is denoted as NBR-PO, the sulfur-cured NBR compound as NBR-Sul, and the sulfur-cured EPDM compound as EPDM-Sul.

### 2.3. 3D Printing

**Figure 1:**
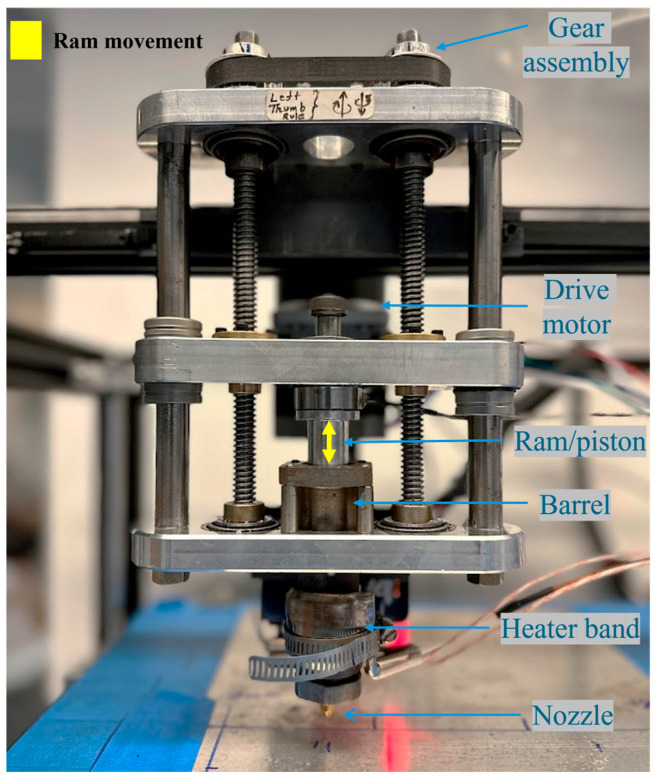
Additive ram material extruder 3XL (ARME-3XL) printhead.

All prints were conducted using a custom print head (shown in Figure 1) mounted on an Ender 5 Pro with modified firmware. The printer (called ARME 3XL) is an updated version of the additive ram material extruder (ARME) discussed in previous work [31] with a stronger stepper motor and lead screws on both sides of the barrel piston setup. The printing process was initiated by loading the rubber compounds, prepared in accordance with Section 2.2, into the barrel of ARME 3XL. The material in the barrel was heated for 5 min under compression, and then printing of the desired geometry was started. All specimens were printed at a 20 mm/sec print speed. The print temperature for NBR-PO and NBR-Sul was kept at 80 °C, and the print temperature for EPDM-Sul was kept at 110 °C to match the viscosity of the rubber formulations during 3D printing. The bed was kept at room temperature. A 0.8 mm nozzle was used, and the layer height was kept constant at 0.3 mm for all prints. Once the printing was completed, parts were removed from the print bed and placed inside an oven for curing. All printed specimens were oven-cured at 160 °C for their respective T90 durations (defined as the time required to reach 90% of the maximum cure, with values reported in Section 3.1.). The curing times were 18 min, 4 min, and 13 min for NBR-PO, NBR-Sul, and EPDM-Sul, respectively. No post-curing treatment was applied to the 3D-printed samples.

### 2.4. Characterization of Rubber Compounds

#### 2.4.1. Cure Behavior and Formulation Viscosity

Rheological and cure behaviors of all rubber formulations were characterized using an oscillating disk curemeter (Rubber Process Analyzer 2000, Alpha technology, Hudson, OH, USA) in accordance with ASTM D2084 [35]. For all rubber compounds, 10 g of uncured rubber specimen was placed inside the RPA, and the cure profile was assessed at the typical print temperature (100 °C) and cure temperature (160 °C). The results of the assessment were presented in Section 3.1. For the viscosity-matching approach discussed in Section 3.1, the cure profiles of NBR-Sul and NBR-PO were characterized at 80 °C, and EPDM-Sul was characterized at 110 °C. The results of this assessment were also presented in Section 3.1.

#### 2.4.2. Post-Print Shrinkage

**Figure 2:**
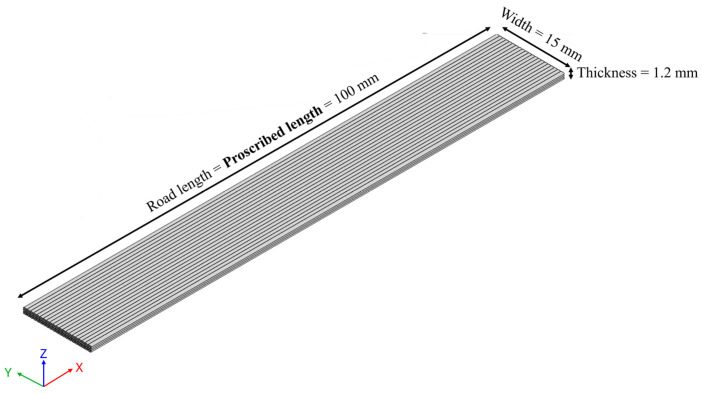
3D model of specimen used for post-print shrinkage measurement with dimensions and print pattern.

Post-print shrinkage refers to the amount of shrinkage the printed parts experience after completion of the print and thermal cure. To quantify the degree of post-print shrinkage, a standard part was printed with each of the three formulations. The part was 100 mm long, 15 mm wide, and 1.2 mm thick. The direction of print was kept aligned to the longest arm of the rectangle, and a regular serpentine print pattern was used. Figure 2 illustrates the dimensions and printing pattern of the part used for post-print shrinkage measurement. Five samples from each compound were printed, and their lengths were recorded at two different stages, namely, after removal of the printed part from the bed and after curing the printed part. Post-print shrinkage was calculated as the percentage of length reduction in the printed parts compared to the proscribed length at various stages mentioned above.

#### 2.4.3. Tensile Behavior

**Figure 3:**
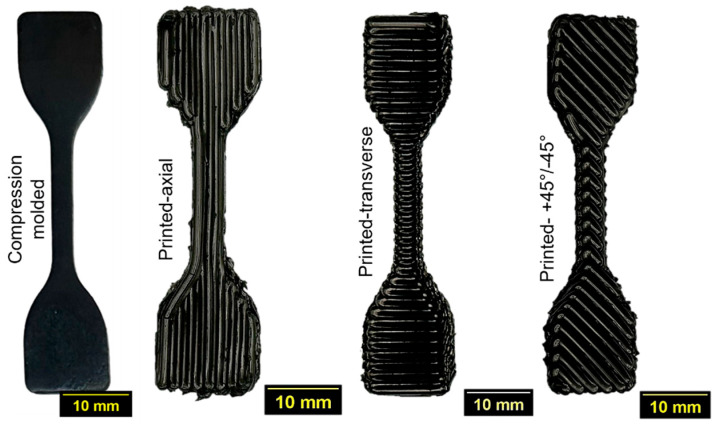
Four types of tensile specimens were tested for each rubber compound. From left to right: compression molded, printed (axial), printed (transverse), and printed (+45°/−45°). Compression-molded samples were die-cut from ASTM D412—Type C tensile specimen at half-scale.

The tensile properties of specimens fabricated from three distinct rubber formulations were evaluated. Three types of printed specimens were assessed: printed (axial), in which the roads were aligned parallel to the direction of applied force; printed (+45°/−45°), where the layer infill angle alternated between +45° and −45° in successive layers; and printed (transverse), in which the roads were oriented perpendicular to the direction of applied force. The 3D-printed tensile specimens were produced to the dimensions of a half-scale ASTM D412-Type C specimen [36]. The tensile properties of these printed specimens were compared with compression-molded (CM) specimens of respective formulations. CM sheets were fabricated using a 152 mm × 152 mm mold cavity with a thickness of 1.5 mm. The CM sheets were cured by maintaining a clamp force of 25 kN at 160 °C for the T90 duration (time required to achieve 90% of the maximum cure) of the respective rubber compound. Tensile specimens were subsequently die-cut from the compression-molded sheets using a half-scale ASTM D412-Type C die. All tensile specimens were tested using a universal testing machine (Instron 4466, Norwood, MA, USA) at a crosshead speed of 500 mm/min, following ASTM D412. An extensometer (Instron 2603-086, Norwood, MA, USA) was employed to record specimen elongation. Figure 3 presents the compression-molded, printed (axial), printed (transverse), and printed (+45°/−45°) samples for comparison.

## 3. Results and Discussion

### 3.1. Rheology and Cure Behavior

**Figure 4:**
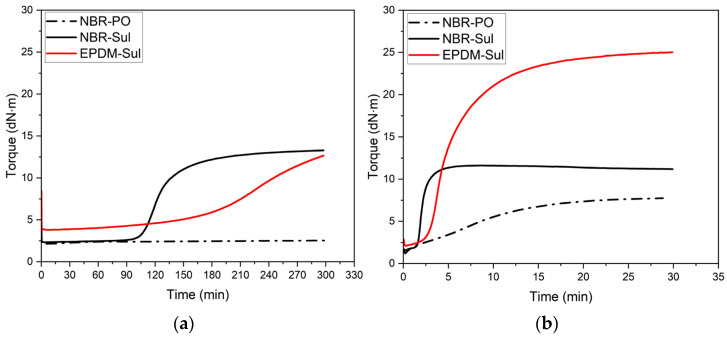
Cure behavior of NBR and EPDM compounds at (**a**) printing temperature of 100 °C and (**b**) oven cure temperature of 160 °C.

**Figure 5:**
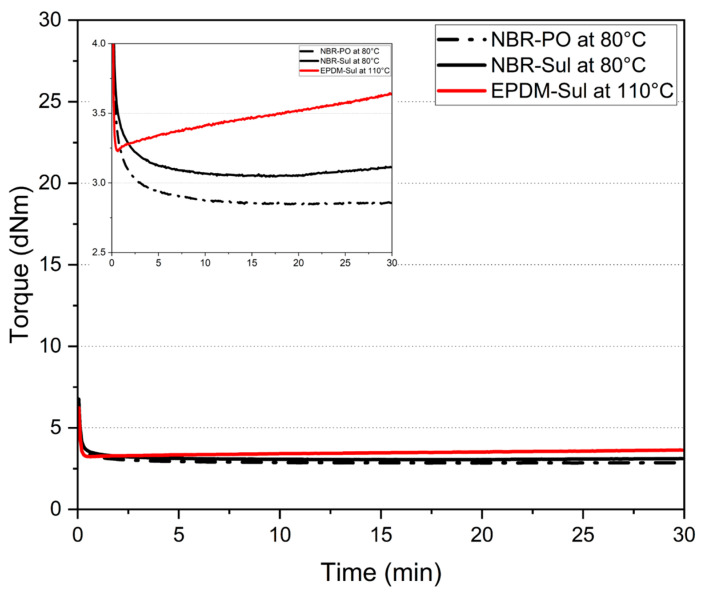
Matching viscosity of NBR and EPDM compounds was achieved by adjusting the processing (printing) temperature. Torque (dN·m) as a function of time (min) is shown for NBR-PO, NBR-Sul, and EPDM-Sul compounds. The inset presents a magnified view of the torque axis (dN·m) versus time (min).

Rubber compounds are thermosetting in nature. When exposed to elevated temperature, the curing process initiates, and irreversible chemical bonds start to form among the base rubber polymeric chains. Such thermosetting nature makes the processing of rubber compounds time and temperature dependent [26,27,28,37,38]. The rubber industry extensively utilizes rubber process analyzers (RPAs), a specific type of oscillating disk rheometer, to monitor the rheological changes experienced by compounded rubber during processing. This instrument functions by measuring the torque required to deform a rubber sample over time, ultimately generating a characteristic “cure curve”. Within the industry, torque values are generally accepted to correlate with the material’s viscosity [39]. Henceforth, NBR and EPDM compound viscosities will be discussed in this study based on the data presented in Figure 4a,b, and Figure 5 with units expressed in decinewton meters (dN·m).

The thermal curing process of compounded rubbers comprises of three distinct phases: the induction or pre-cure phase, the curing phase, and lastly, the over-cure phase [40]. The initial stage of thermal cure, known as the induction phase, starts when the rubber compounds are exposed to an elevated temperature. During this phase, the formation of crosslinks begins, leading to a moderate rise in the compound’s viscosity. However, the rubber compound remains processable and exhibits characteristics similar to a fluid throughout this induction phase [26,27,28,37,38]. 3D printing of all rubber samples was conducted at this induction phase. The duration of the induction phase (processing window) is denoted as scorch time (ts2), the time required for the state of cure to increase two torque units above the minimum torque (ML) at the given cure temperature [40]. After ts2, it is considered that the rubber compound can no longer be processed or formed into shape. Since viscosity is a critical factor influencing the printability and part properties of fully compounded thermoset elastomers [34,41], evaluating the compound’s viscosity at the printing temperature during this stage is essential. Following the initial pre-cure stage, the curing phase takes place. This stage is characterized by a rapid increase in crosslink formation within the rubber compound matrix. This rapid crosslinking is reflected by a swift rise in the measured torque values [26,27,28,37,38]. As curing progresses, a plateau region is reached, indicating that the optimal cure state has been achieved. In the rubber industry, the optimum cure time (t_90) is defined as the time required for the torque to reach 90% of the difference between the maximum torque (MH) and the minimum torque (ML). At t_90, the final product attains the desired physical properties necessary for optimal performance. Terminating the heating process at t_90 helps to prevent over-curing or reversion, both of which can negatively impact the final product’s properties [40].

Figure 4a shows the rheological and cure behavior of rubber compounds at 100 °C, a typical print temperature in rubber 3D printing [34]. Figure 4b shows the rheological and cure behavior of rubber compounds at 160 °C, the cure temperature of printed parts. Cure parameters such as minimum torque (ML), maximum torque (MH), scorch time (ts2), cure time (t90), and maximum torque difference (related to crosslink density) (MH-ML) are reported in Table 2. It is evident from Figure 4a and Table 2 that under similar processing temperatures, the EPDM-Sul compound showed the highest viscosity with an ML value of 3.8 dN·m. On the other hand, both NBR compounds, NBR-PO and NBR-Sul, showed 40–44% lower viscosity with ML values of 2.1 dN·m and 2.3 dN·m, respectively.

In previous works, we showed that the viscosity of rubber compounds at the print temperature plays a critical role in determining the post-print shrinkage and adhesion quality among the printed part’s neighboring roads [34]. In order to neutralize the effects of the rubber compound’s viscosity and evaluate the effects of different components of the rubber formulation on the post-print shrinkage and part properties, a viscosity-matching approach was taken. The viscosity of the NBR and EPDM compounds was brought very close to each other by changing their respective processing temperatures. Figure 5 shows the outcome of the viscosity-matching process by temperature alteration. By lowering the processing temperature for the NBR-PO and NBR-Sul compounds to 80 °C and raising the processing temperature for the EPDM-Sul compound to 110 °C, the gap between the ML values of the rubber compounds was brought down to a comparable level. Based on this, a printing temperature of 80 °C was assigned to the NBR-PO and NBR-Sul compounds, and a printing temperature of 110 °C was assigned to the EPDM-Sul compound.

#### 3.1.1. Post-Print Shrinkage

**Figure 6:**
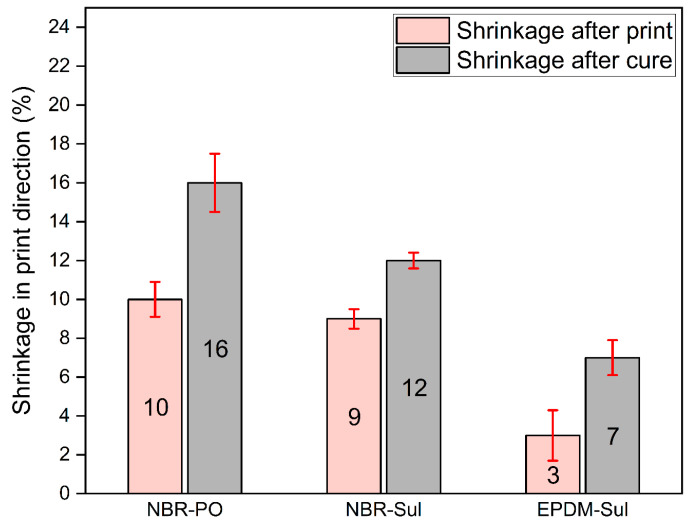
Shrinkage of 3D-printed rubber compound parts in the print direction after completion of print and after completion of thermal cure.

Post-print shrinkage refers to the amount of shrinkage the printed parts experience after completion of the print and thermal cure. Figure 6 shows the post-print and post-cure shrinkage of the printed parts from NBR and EPDM compounds. Previous research identified post-print shrinkage as a significant hurdle in using material extrusion additive manufacturing (MEx AM) for fabricating parts with fully compounded rubbers or thermoset elastomers [31]. This phenomenon arises from the inherent tendency of polymer chains to relax back to their original, coiled state after being stretched [42]. During the printing process, rubber compounds are forced through the narrow opening of the printer nozzle. This process applies stress to the polymer chains, causing them to elongate. Once deposited on the print bed, the stress on the extruded rubber compound gradually relaxes due to the viscoelastic properties of rubber. As this stress dissipates, the elongated polymer chains return to their coiled configuration. This behavior of the polymer chains is the underlying cause of post-print shrinkage observed in MEx AM of rubber compounds.

Both peroxide- and sulfur-cured NBR formulations showed very similar values of shrinkage after print: 10% and 9%, respectively. Notably, their standard deviations overlapped, indicating statistically similar initial shrinkage. However, after thermal curing, the NBR-PO formulation exhibited higher shrinkage (16%) compared to the NBR-Sul formulation (12%). Such shrinkage behavior of rubber compounds as a function of the cure system can be attributed to the nature of crosslinks that each of the curing systems generates. Since both NBR formulations possessed comparable viscosities at the printing temperature and were printed at identical extrusion rates, their initial post-printing shrinkage values were statistically indistinguishable. However, during the curing process, the peroxide-cured systems generate C-C bonds as crosslinks, and sulfur-cured systems generate alkyl-C-S-C-alkyl bonds. The C-C crosslinks formed during peroxide curing are more rigid and stronger than the sulfur crosslinks formed during sulfur curing [26]. Compared to the more flexible sulfur crosslinks, the stronger and more rigid C-C bonds might have caused faster retraction of the elongated polymeric chains of the extruded compounds to their initial state of relaxation and thus might have caused the greater shrinkage measured after the completion of the thermal curing process.

Figure 6 also shows that the EPDM-Sul compound showed threefold lower shrinkage after printing and about twofold lower shrinkage after curing compared to the NBR-Sul compound. Such behavior can be explained by the comparative elastic behavior of NBR and EPDM rubbers. Tiwari et al. [43] evaluated the storage modulus of sulfur-cured NBR and sulfur-cured EPDM compounds under tension and found that at any particular temperature, the storage modulus of sulfur-cured NBR is greater than that of sulfur-cured EPDM compounds at all tested frequencies. This suggests greater elasticity of NBR compared to EPDM under similar formulations and test conditions. The greater elastic nature of NBR stock polymer might have caused faster retraction of the elongated polymeric chains of the extruded compounds to their initial state of relaxation and thus might have caused the greater post-print and post-cure shrinkage.

#### 3.1.2. Available Processing Window

As discussed in Section 3.1, the thermosetting nature of rubber compounds makes rubber processing time and temperature dependent. At any processing temperature, the rubber compound only remains processable during the pre-cure or induction phase. The duration of the pre-cure phase is called scorch time (ts2). Thus, in rubber 3D printing, the scorch time of any rubber compound becomes an important parameter denoting the available printing window before the compound becomes processable. Besides temperature, the scorch time of any rubber compound also depends on the curing agent type and content, among other formulation ingredients. Section A of Table 2 reports the scorch time of the rubber compounds used in this study at a conventional printing temperature of 100 °C [31,34]. Since both the NBR formulations are the same except for the type of curing agent used, Table 2 gives a comparative evaluation of the suitability of the cure system in 3D printing applications from the point of view of the available printing window. Since AM is characteristically a slow manufacturing process, often taking hours to days to complete a single part, a lengthy processing window allows the formulation to be used for printing of large parts.

At 100 °C, the peroxide-cured NBR shows a scorch time of more than five hours. Even at the end of the fifth hour, the NBR-PO formulation showed no sign of curing, giving the formulation a very wide processing window and making the formulation suitable for 3D printing of large parts. On the other hand, the NBR-Sul formulation showed a scorch time of about three hours. Hence, the NBR-Sul formulation should be suitable for 3D printing parts requiring less than three hours of print time.

### 3.2. Tensile Behavior

**Figure 7:**
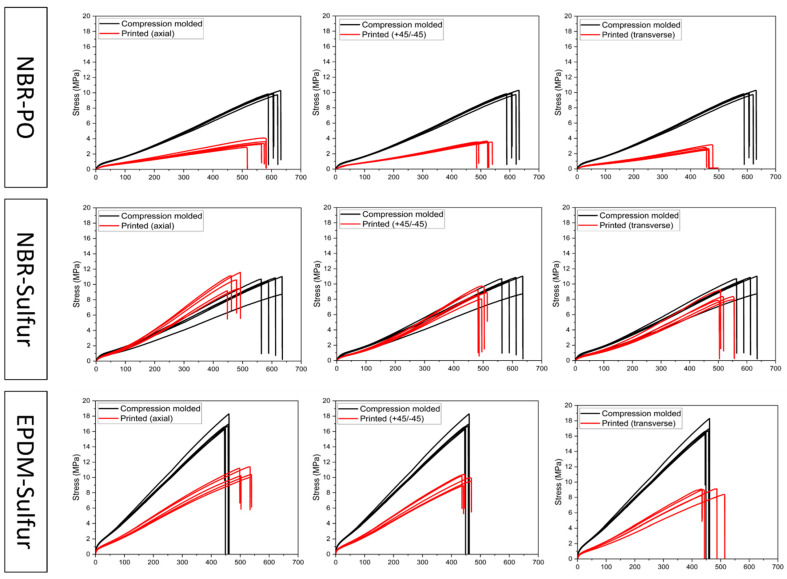
Stress–strain behavior of NBR-PO, NBR-Sul, and EPDM-Sul (**top** to **bottom**, respectively) printed parts in axial (**left**), +45°/−45° (**middle**), and transverse (**right**) directions of applied force.

Five replicates for each type of tensile specimen, namely printed (axial), printed (+45°/−45°), printed (transverse), and compression molded (CM), were prepared from each rubber compound. These samples were then subjected to tensile testing. Figure 7 shows the stress–strain curves of the 3D-printed samples benchmarked against respective compression-molded samples from each formulation. A table summarizing the key tensile properties (ultimate tensile stress, strain at break, modulus, normalized stress at break, and anisotropy ratio) for all tested specimens is provided in Table 3. Due to the non-linear nature of elastomers, the modulus was calculated at 100% elongation, following a common practice for elastomeric materials [44,45]. Moreover, the dependence of tensile properties on the raster orientation of printed specimens (anisotropy) was evaluated. Raster orientation-induced anisotropy was quantified using the anisotropy ratio, defined as the ratio of the minimum to the maximum value of stress or strain at break. Anisotropy ratio is a dimensionless parameter ranging from 0 to 1, where values closer to 1 indicate greater isotropy in mechanical behavior [46,47]. These anisotropy ratios were then compared across formulations to assess the relative degree of anisotropy associated with each rubber compound.

For each formulation, compression-molded specimens exhibited the highest tensile strength and elongation, while 3D-printed specimens showed reduced absolute strength due to interlayer interfaces. Such behavior is a common phenomenon in additive manufacturing due to the presence of voids at the layer-to-layer interfaces. Among the 3D-printed samples, sulfur-cured NBR and EPDM exhibited comparable tensile strengths, whereas peroxide-cured NBR showed significantly lower tensile strength across all printing orientations. For elongation at break, 3D-printed samples from all compounds showed high elongation, ranging from 450 to 560%, depending on print orientation. Additionally, the tensile behavior of all three rubber compounds was found to be strongly dependent on printing orientation, showing the anisotropic behavior typical of extrusion-based additive manufacturing. Sulfur-cured NBR showed the least anisotropy for stress at break (0.82) and strain at break (0.90), whereas peroxide-cured NBR showed the highest anisotropy in stress (0.74) and strain (0.82). The anisotropy ratios for sulfur-cured NBR and EPDM compounds were very similar for stress (0.82 vs. 0.82) and comparable for strain (0.90 vs. 0.87).

**Figure 8:**
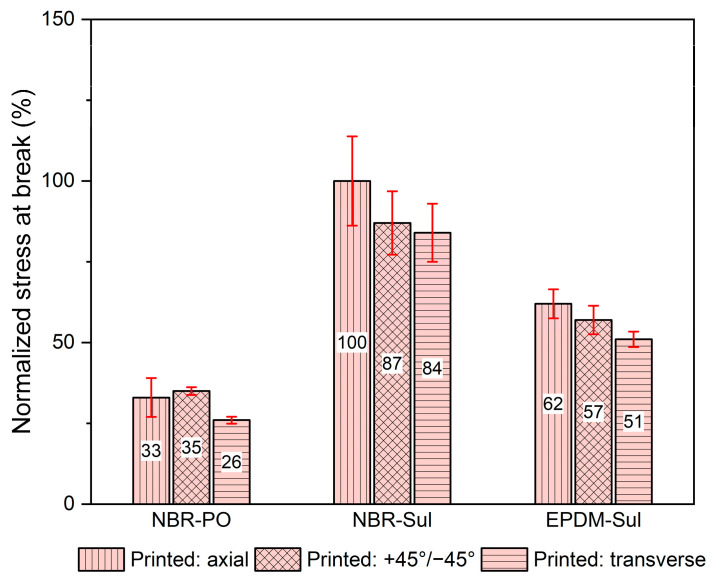
Normalized stress at break of 3D-printed samples.

As observed in Figure 7 and Table 3, the specific formulation significantly impacted the ultimate tensile stress of the compression-molded samples. This variation in the base material properties (due to formulation) could lead to corresponding variations in the tensile properties of the printed samples. Recognizing the potential influence of formulation on tensile properties, a direct comparison of the ultimate tensile stress of printed samples was avoided. This approach would not provide an accurate assessment of the comparative strength and quality of interlayer adhesion between the rubber formulations. Hence, a new parameter, normalized stress at break, was introduced to address this challenge. This normalized stress was calculated as the ratio of the ultimate tensile stress obtained from a printed sample to the ultimate tensile stress of its corresponding compression-molded sample. Normalized stress at break provides an indication of the degree of adhesion between neighboring printed roads. It quantifies the percentage of bulk material strength retained by the 3D-printed samples relative to their corresponding compression-molded counterparts. This approach allowed for a more focused evaluation of the interlayer adhesion strength between the printed layers. The normalized stress at the break of the printed samples was calculated and plotted in Figure 8.

3D-printed samples from NBR-Sul exhibited superior performance compared to EPDM-Sul in terms of normalized stress at break across all printing orientations. Notably, axially printed NBR-Sul specimens (printed layers parallel to the tensile force) displayed an impressive 100% normalized stress at break, indicating that they retained 100% strength of the respective compression-molded samples. Similarly, transversely printed NBR-Sul specimens (printed layers perpendicular to the force) achieved a noteworthy 84% normalized stress at break. Specimens printed at a 45-degree angle (“Printed: +45°/45° NBR-Sul”) exhibited 87% normalized stress at break, falling between axially and transversely printed samples. These high and comparable normalized stress at break values across different printing orientations suggest good isotropy in the NBR-Sul printed samples. In contrast, EPDM-Sul printed specimens in axial, +45°/−45°, and transverse orientations exhibited significantly lower normalized stress at break values of 62%, 57%, and 51%, respectively. This implies that EPDM-Sul printed parts retained only about half the strength of their compression-molded counterparts. It also suggests weaker adhesion between neighboring printed layers compared to NBR-Sul.

While previous research has shown viscosity to be a critical factor in interlayer adhesion [31,34,42], in this case, viscosity was kept constant via the viscosity-matching approach. However, the differences in normalized stress at break might be due to differences in their intrinsic molecular properties, such as molecular weight, microstructure, crystallinity, and polarity [48]. It is well documented in the rubber literature that NBR generally exhibits great self-adhesion due to its polar nature [49,50,51]. Voiutskii [50] reported that polar elastomers, such as NBR, exhibit better self-adhesion due to stronger interfacial forces arising from polar interactions between polymer chains at the interface. This behavior may contribute to the excellent adhesion and high strength retention observed in NBR-Sul samples across all printing orientations. In contrast, the relatively poor self-adhesion of EPDM compounds is well documented in the rubber literature, and EPDM formulations often require tackifiers to achieve sufficient self-adhesion [48,52,53]. The absence of a tackifier in the EPDM compound used in this study may therefore have contributed to the lower degree of adhesion between neighboring printed layers. Ruch et al. attributed the insufficient self-adhesion of EPDM to its branching and high entanglement density, which could drastically reduce chain mobility across the interface [52]. Moreover, Bothe et al. [54] found that crystallites present in unstretched EPDM hinder chain mobility. Although the degree of crystallinity of the EPDM rubber used in this study was not evaluated, it may represent an additional contributing factor to the reduced interlayer adhesion observed in the printed EPDM samples.

3D-printed samples made from NBR-PO exhibited significantly lower normalized stress at break compared to the excellent normalized stress at break values of their NBR-Sul counterparts across all printing orientations (axial, +45°/−45°, and transverse). Notably, NBR-PO retained only about one-third or less of the strength of compression-molded samples (33%, 35%, and 26%, respectively). Since both NBR-Sul and NBR-PO formulations had similar viscosity, the disparity in their normalized stress at break values can be directly associated with the variance in their curing agents (sulfur vs. peroxide)—the sole distinguishing factor in the formulations. During the curing process, the peroxide-cured systems generate C-C bonds as crosslinks, and sulfur-cured systems generate alkyl-C-S-C-alkyl bonds. The C-C crosslinks formed during peroxide curing are more rigid and stronger than the sulfur crosslinks formed during sulfur curing [26]. The flexible sulfur crosslinks in NBR-Sul might have allowed for better interlayer diffusion of polymer chains than the stronger and more rigid C-C bonds in NBR-PO. This enhanced diffusion during printing could potentially facilitate stronger adhesion between adjacent printed layers. Consequently, NBR-Sul printed parts exhibit superior normalized stress at break, indicating better preservation of bulk material strength than NBR-PO printed parts.

## 4. Conclusions

This study evaluated the effects of two critical ingredients of a rubber compound—base rubber (NBR vs. EPDM) and curing agent (sulfur vs. peroxide)—on printed parts’ post-print shrinkage and tensile behavior (interlayer adhesion). An NBR and an EPDM formulation were designed to explore the impacts of base rubber with identical ingredients except for the base elastomer. In addition, a sulfur-cured and a peroxide-cured NBR formulation were also designed with identical formulation ingredients except for the curing agent.

A comparative assessment between the NBR and EPDM formulations revealed that when printing parameters and formulation viscosity were kept constant, printed samples from sulfur-cured EPDM exhibited lower post-print and post-cure shrinkage but retained only 51–62% bulk strength. Conversely, printed samples from sulfur-cured NBR displayed higher shrinkage but achieved 84–100% strength retention.

A comparison between sulfur- and peroxide-cured NBR formulations showed that when printing parameters and formulation viscosity were kept constant, printed samples from both formulations showed statistically identical shrinkage immediately after the completion of printing. However, the peroxide-cured NBR formulation showed higher shrinkage (16%) after thermal cure than the sulfur-cured NBR formulation (12%). Moreover, the peroxide-cured NBR formulation also showed substantially lower normalized stress at break (26–33%) than the sulfur-cured NBR formulation (84–100%). Notably, the peroxide cure system provided almost twice as much processing window as the sulfur cure system.

Proper design of a rubber compound for any processing technique requires detailed characterization of formulation effects on the processability and properties of the final product. Given the inherent similarities in formulation ingredient classes and their intended functionalities across various traditional rubber compounds, this study holds the prospect of guiding future formulation development with various synthetic rubbers, such as BR, SBR, HNBR, EPDM, FKM, and FFKM, for 3D printing applications. This study also shows prospects of additively manufactured parts with tailored properties for various industrial applications.

## Figures and Tables

**Table 1:** Formulation of all rubber compounds.

Element	Component	Mix Time (min)	Parts Per Hundred Rubber (PHR)		
			NBR-PO	NBR-Sul	EPDM-Sul
Elastomer	High MW NBR	1	50	50	0
	Low MW NBR	1	50	50	0
	High MW EPDM	1	0	0	50
	Low MW EPDM	1	0	0	50
Filler	Carbon black	5	60	60	60
Activators	Zinc oxide	1	4	4	4
	Stearic acid	1	0.5	0.5	0.5
Accelerators	CBS	1	0	2	2
Vulcanizing agent	Sulfur	10	0	1	1
	Peroxide (PO)	10	1	0	0

**Table 2:** Cure parameters of rubber compounds at print (100 °C) and cure (160 °C) temperatures.

	NBR-PO	NBR-Sul	EPDM-Sul
A. 240 min at 100 °C			
Minimum torque, ML (dN·m)	2.1	2.3	3.8
Maximum torque, MH (dN·m)	2.5	13	13
Scorch time, ts2 (min)	300+	175	276
B. 60 min at 160 °C			
Minimum torque, ML (dN·m)	1.3	1.5	2.1
Maximum torque, MH (dN·m)	7.8	11.2	25
Cure time, t90 (min)	18	4	13
Crosslinking density, MH-ML (dN·m)	6.5	9.7	22.9

**Table 3:** Mean ± SD of tensile properties of tested samples, including stress at break, strain at break, modulus at 100% strain, and normalized stress at break.

	NBR-PO	NBR-Sul	EPDM-Sul
A. Stress at break (MPa)			
Compression molded	10 ± 0.2	10.1 ± 0.9	17.2 ± 0.8
Printed (axial)	3.3 ± 0.6	10.4 ± 1.1	10.6 ± 0.6
Printed (+45°/−45°)	3.5 ± 0.1	8.8 ± 0.6	9.8 ± 0.6
Printed (transverse)	2.6 ± 0.1	8.5 ± 0.5	8.7 ± 0.1
B. Strain at break (%)			
Compression molded	610 ± 16	606 ± 31	454 ± 7
Printed (axial)	563 ± 27	474 ± 20	521 ± 20
Printed (+45°/−45°)	512 ± 23	494 ± 14	451 ± 16
Printed (transverse)	463 ± 8	525 ± 25	460 ± 36
C. Modulus at 100% strain (MPa)			
Compression molded	1.7 ± 0.1	1.8 ± 0.2	4.3 ± 0.1
Printed (axial)	0.8 ± 0.1	1.7 ± 0.2	2.4 ± 0.2
Printed (+45°/−45°)	0.8 ± 0.1	1.4 ± 0.1	2.4 ± 0.2
Printed (transverse)	0.7 ± 0.1	1.3 ± 0.1	2.1 ± 0.2
D. Normalized stress at break (%)			
Compression molded	100	100	100
Printed (axial)	33	100	62
Printed (+45°/−45°)	35	87	57
Printed (transverse)	26	84	51
E. Anisotropy ratio			
Stress at break	0.74	0.82	0.82
Strain at break	0.82	0.90	0.87

## Data Availability

The data presented in this study are contained within the article. Further inquiries can be directed to the authors.
